# Prioritized nonlinear modeling of shared dynamics across neural populations

**DOI:** 10.1088/1741-2552/ae9bee

**Published:** 2026-10-16

**Authors:** Trisha Jha, Dong Song, Maryam M Shanechi

**Affiliations:** 1 Ming Hsieh Department of Electrical and Computer Engineering, Viterbi School of Engineering, University of Southern California, Los Angeles, CA, United States of America; 2 Department of Neurological Surgery, University of Southern California, Los Angeles, CA, United States of America; 3 Alfred E. Mann Department of Biomedical Engineering, University of Southern California, Los Angeles, CA, United States of America; 4 Thomas Lord Department of Computer Science, Neuroscience Graduate Program, University of Southern California, Los Angeles, CA, United States of America

**Keywords:** multiregional modeling, nonlinear dynamical models, RNNs, filtering, smoothing

## Abstract

*Objective*. Advances in neural recording technology enable simultaneous measurements across multiple brain regions, providing new opportunities to study inter-regional interactions. However, several challenges arise when developing nonlinear dynamical models of cross-region interactions. In particular, we identify three key desired properties for a nonlinear framework. First, it should prioritize extraction of cross-region dynamics to avoid confounds from within-region dynamics. Second, it should enable localization of nonlinear structure within the model to better interpret cross-regional interaction models. Third, it should support both temporally causal and non-causal inference of shared dynamics and do so using source region activity alone. Current cross-regional models do not satisfy all these properties. *Approach*. Here, we address these challenges by developing cross-population prioritized dynamical nonlinear interaction model (CroP-DYNO), a nonlinear dynamical framework that prioritizes the learning of shared cross-population dynamics to avoid confounds from within-population activity. Further, CroP-DYNO allows individual components of the dynamical model to be independently specified as nonlinear or linear. Finally, it supports both temporally causal and non-causal inference of latent states, using only source region activity. *Main results*. We validate our method on datasets across species and distinct brain regions. We find that both the prioritized learning and the nonlinear modeling in CroP-DYNO are important for accurately extracting cross-population dynamics. As such, CroP-DYNO outperforms baseline nonprioritized and linear prioritized models in predicting target neural population activity from source activity. Further, CroP-DYNO enables systematic localization of nonlinear structure and quantifies dominant predictive interactions between brain regions, with interaction strengths that align with known circuit anatomy. *Significance*. Overall, these results establish CroP-DYNO as a flexible nonlinear framework for studying interactions between neural populations and brain regions, enabling both accurate modeling and interpretable dissection of nonlinear structure in cross-regional communication models.

## 1. Introduction

Understanding how different brain regions communicate is a fundamental problem in neuroscience, as most cognitive, sensory, and motor functions emerge from coordinated activity across multiple regions [1–7]. Characterizing this communication requires quantifying both the directionality and strength of the interaction across regions. Recent advances in large-scale neural recording technologies have made this goal increasingly tractable. In particular, the growing availability of simultaneous multi-region recordings now enables the study of interactions across brain regions [8–15].

Many previous studies of cross-region interactions have relied on linear methods [16–23]. Linear approaches offer many important advantages: they are computationally efficient, interpretable, and often robust in data-limited regimes. As a result, these methods have provided valuable insights into how brain regions communicate to support behavior. However, linear models also face some limitations. A first concern is biological plausibility. Many neural systems, such as the somatosensory or motor system and hippocampus, exhibit nonlinearity in their activity or interactions [24–28]. Indeed, nonlinear dynamics in neural systems may underlie key neural functions, including neuronal encoding and communication between neural populations [29, 30]. Second, linear models may not be sufficiently expressive in capturing neural dynamics and interactions [31–33], whereas nonlinear models may better account for additional structure and interactions in neural data [27, 28, 34–39]. Together, these observations motivate the development of nonlinear approaches to better characterize the interactions across brain regions [29, 40–42].

Moving beyond linearity, however, introduces new modeling challenges. In particular, we identify three key desired properties for a nonlinear framework aimed at characterizing cross-region interactions.

First, the framework should prioritize the extraction of shared cross-region dynamics during modeling, such that they are not masked or confounded by within-region dynamics. This is because in multi-region recordings, the activity measured in each region reflects a mixture of dynamics shared across regions and dynamics local to that region. In this work, shared cross-population dynamics refer to source-derived target-predictive dynamics, namely the latent dynamics in a source population that help predict the target population, suggesting cross-population interactions. Indeed, recent work in the linear setting has shown that explicitly prioritizing cross-region prediction can mitigate this confound [22], but this prioritization principle has not yet been extended to the nonlinear cross-region modeling regime.

Second, to enable interpretability, the framework should enable localization of nonlinear transformations within specific components of the cross-region mapping. Indeed, although fully nonlinear models implemented with deep learning may achieve strong predictive performance, their increased flexibility can obscure where nonlinear transformations arise. When the goal is not only to predict neural activity but also to understand how information is transformed as brain regions communicate [40], it is important to determine which aspects of cross-region mappings are nonlinear rather than artifacts of the model structure.

Third, the framework should support both causal and non-causal inference, the former providing more interpretable analyses and the latter allowing for more accurate inference of shared dynamics. We note that throughout this work, ‘causal’ and ‘non-causal’ refer to the temporal nature of inference, namely whether inference uses only past or also future source activity, rather than directed causality. In particular, causal (filtering) inference, which uses only past source-region activity, aids interpretability by ensuring that predicted signals in the target region appear first in the source, thus enabling analyses of directed interactions. Non-causal (smoothing) inference, which also incorporates future source activity, can improve latent state estimation in noisy data. This benefit has also been shown within the linear setting [22]. A flexible framework should accommodate both causal and non-causal inference depending on the analysis goals. Additionally, in both modes, the framework should support inference of shared dynamics using only source region activity, without requiring access to target region recordings at inference time. This capability is important for studying directed source-target interactions [22] and for practical settings such as brain-computer interfaces and neuromodulation systems, where target region recordings may be unavailable [27, 33, 43–50].

To date, no framework for multi-region modeling satisfies these three desired properties. In this work, we address these challenges by developing the cross-population prioritized dynamical nonlinear interaction model (CroP-DYNO). Given neural activity from two populations, CroP-DYNO learns a nonlinear dynamical model in the form of recurrent neural networks (RNNs) that explicitly prioritizes the extraction of cross-population dynamics over within-population dynamics. This is achieved by designing a learning objective that optimizes accurate prediction of target population activity from source population activity. The framework also localizes nonlinear transformations by allowing individual components of the model, such as the source latent dynamics or the mapping from source latent states to target neural activity, to be specified as either linear or nonlinear. This enables the testing of hypotheses about which components of cross-region communication are nonlinear. Finally, CroP-DYNO supports inference of shared dynamics both causally and non-causally in time and using source region activity alone. CroP-DYNO follows a numerical optimization approach similar to [28] for modeling behaviorally relevant neural dynamics but reformulates it for extracting cross-population dynamics as a prioritized learning problem with a cross-region prediction objective. Further, unlike [28] which only supports causal inference, CroP-DYNO also supports non-causal inference of cross-region dynamics.

We validate CroP-DYNO as a useful tool for modeling cross- and within-region neural dynamics across multiple neural datasets spanning species and brain systems. In particular, we examine somatosensory–motor cortical interactions in a non-human primate (NHP) and hippocampal CA1–CA3 interactions in a rat. To model cross-region dynamics, we use CroP-DYNO to model shared dynamics between neural populations drawn from different brain regions. To model within-region dynamics, we use CroP-DYNO to model shared dynamics between two non-overlapping populations within that region. Across datasets, we show that prioritized learning is important for extracting cross-region dynamics by comparing CroP-DYNO with a nonlinear RNN without prioritization and with a recent cross-region modeling method based on Gaussian processes that does not aim for prioritization [51]. In addition to prioritization, we also demonstrate that nonlinear modeling is important for cross-region analyses by comparing with a recent linear prioritized cross-region model (CroP-LDM) [22]. Furthermore, by selectively setting individual model components to be linear or nonlinear, CroP-DYNO localizes where nonlinear structure arises in the cross-population model. Lastly, the framework can quantify dominant predictive interactions between brain regions with interaction strengths that align with known circuit anatomy. Together, these results establish CroP-DYNO as a flexible framework for studying nonlinear interactions across neural populations.

## 2. Methods

### 2.1. Neural recordings, behavioral tasks, and data preprocessing

The neural data analyzed in this study span different brain systems and animal models. The first dataset was collected and made publicly available by the Sabes lab [52]. In this dataset, Monkey I controlled a cursor based on fingertip position on a 2D surface in a 3D virtual reality environment. Neural activity from the primary somatosensory cortex S1 and the primary motor cortex M1 was recorded using 96-electrode microelectrode arrays. All animal procedures were performed in compliance with the National Research Council Guide for Care and Use of Laboratory Animals, and were approved by the Institutional Animal Care and Use Committee at University of California San Francisco. We analyzed data for the first 7 recording sessions available (sessions 20160407/01–20160426/01).

The second dataset consisted of a male Long-Evans rat trained on a two-lever spatial delayed-non-match-to-sample task with random delay intervals [53, 54]. Neural activity from both hemispheres of hippocampal regions CA1 and CA3 was recorded using a microwire multi-electrode array. All animal procedures were reviewed and approved by the Institutional Animal Care and Use Committee of Wake Forest University, in accordance with US Department of Agriculture, International Association for the Assessment and Accreditation of Laboratory Animal Care and National Institutes of Health guidelines. We analyzed data from 3 recording sessions.

In our analyses, we modeled neural spiking activity. Specifically, we counted spikes in 10 ms non-overlapping bins, smoothed the resulting spikes counts with a Gaussian kernel with standard deviation 50 ms [55–58], and downsampled to a 50 ms time step.

### 2.2. CroP-DYNO algorithm

#### 2.2.1. Model formulation

We present the CroP-DYNO framework and describe how it can be used to study interactions across neural populations. CroP-DYNO models the neural activity nonlinearly as the below dynamical system model (figure 1):

\begin{equation*}\left\{ {\begin{array}{*{20}{c}} {{x_{k + 1}} = {A^{\prime} }\left( {{x_k}} \right) + K\left( {{a_k}} \right)} \\ {{a_k} = {C_a}\left( {{x_k}} \right) + {e_k}} \\ {{b_k} = {C_b}\left( {{x_k}} \right) + { \epsilon _k}} \end{array},{\text{ }}{x_k} = \left[ {\begin{array}{*{20}{c}} {x_k^{\left( 1 \right)}} \\ {x_k^{\left( 2 \right)}} \end{array}} \right]} \right.\end{equation*}

**Figure 1.**
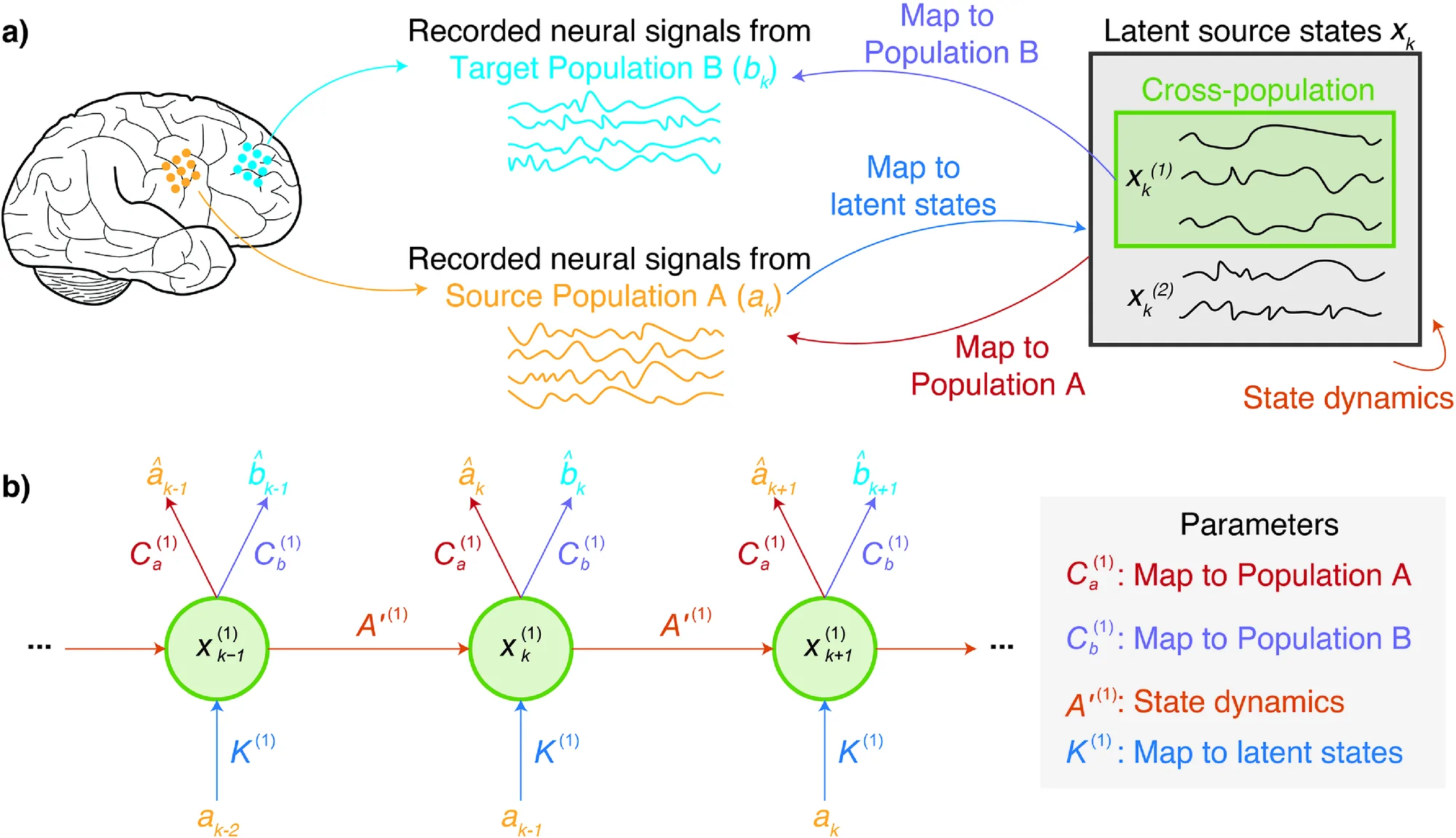
Overview of the CroP-DYNO framework. (a) Neural activity is recorded from the source population A (${a_k})$ and target population B $\left( {{b_k}} \right)$. CroP-DYNO models the underlying neural dynamics using a latent state composed of two components: $x_k^{\left( 1 \right)}$, the dynamics shared between populations A and B, and, the within-population A dynamics not shared with population B. The model comprises four mappings: latent state dynamics (*A*’), a mapping from source population A activity to latent states (*K*), and mappings from latent states to activity from populations A (${C_a}$) and B (${C_b}$). (b) The CroP-DYNO computation graph for the shared cross-population states $x_k^{\left( 1 \right)}$ represented as an RNN, with parameters corresponding to the mappings introduced in a. At each time step, the RNN receives the activity of source population A as input and outputs predictions of the activity of populations A and B at the next time step. The superscripts (1) indicate that the parameters are associated with the shared cross-population states $x_k^{\left( 1 \right)}$,

where ${a_k} \in {\mathbb{R}^{{n_a}}}$ and ${b_k} \in {\mathbb{R}^{{n_b}}}$ are the recorded activity in neural populations A and B, respectively. The latent state ${x_k} \in {\mathbb{R}^{{n_x}}}$ captures the latent dynamics underlying population A and is composed of sections $x_k^{\left( 1 \right)} \in {\mathbb{R}^{{n_1}}}$ and $x_k^{\left( 2 \right)} \in {\mathbb{R}^{{n_2}}}$ (with ${n_2} = {n_x} - {n_1}$). Specifically, models the dynamics of population A that are shared with population B (i.e. cross-population dynamics), while $x_k^{\left( 2 \right)}$ models the within-population A dynamics that are not shared with population B (figure 1(a)). The terms ${e_k}$ and ${ \epsilon _k}$ represent observation noises.

The functions $A^{\prime}$, K, ${C_a}$, and ${C_b}$ are multi-input, multi-output functions that parametrize the state dynamics, mapping from population A to latent state ${x_k}$, and mappings from the latent state ${x_k}$ to populations A and B, respectively (figure 1(a)). In this work, our main goal is to learn the shared cross-population latents $x_k^{\left( 1 \right)}$ and their associated parameters.

#### 2.2.2. Learning the model parameters

Given training data, we adopt a prioritized learning strategy that learns parameters associated with the shared cross-population states, $x_k^{\left( 1 \right)}$, without the need to learn the model parameters associated with $x_k^{\left( 2 \right)}$. We take this prioritized approach because it allows us to fit a low-dimensional latent dynamical model, where the total state dimension is the dimension of $x_k^{\left( 1 \right)}$ rather than the total dimension of ${\text{ }}{x_k}$. This low-dimensional state is explicitly trained to capture the cross-population dynamics—the dynamics in population A that are shared with population B—using a cross-population prediction objective described below.

We can apply this prioritized learning approach to investigate both cross-region and within-region interactions. Specifically, taking ${a_k}$ and ${b_k}$ as non-overlapping populations drawn from different brain regions yields a cross-region model, while drawing both from the same region yields a within-region model (details in section 2.6). This approach avoids the need for learning $x_k^{\left( 2 \right)}$ and its associated model parameters.

The learning proceeds as follows. We first learn the shared cross-population latent states $x_k^{\left( 1 \right)}$ and their corresponding parameters ${A^{{\prime ^{\left( 1 \right)}}}}$, ${K^{\left( 1 \right)}}$, and $C_b^{\left( 1 \right)}$ within the below dynamical model:

\begin{equation*}\left\{ {\begin{array}{*{20}{c}} {x_{k + 1}^{\left(1\right)} = A^{^{\prime}{\left(1\right)}}\left(x_k^{\left(1\right)}\right) + {K^{\left(1\right)}}\left({a_k}\right)} \\ {{b_k} = C_b^{\left(1\right)}\left(x_k^{\left(1\right)}\right) + { \epsilon _k}} \end{array}} \right.\end{equation*}

This dynamical model represents an RNN with ${A^{{\prime ^{\left( 1 \right)}}}}$, ${K^{\left( 1 \right)}}$, and $C_b^{\left( 1 \right)}$ being nonlinear transformations, which we implement using feedforward neural networks (figure 1(b)). This model describes the temporal evolution and mappings of the shared cross-population states, as also indicated by the superscript (1). To ensure that $x_k^{\left( 1 \right)}$ captures the dynamics shared between source and target populations, we learn the model parameters to optimize the prediction of target activity from source activity. Specifically, given training source and target neural activity ${a_k}$ and ${b_k}$, we devise the following cross-population prediction objective

\begin{equation*}{L_b} = \mathop \sum \limits_k\Big \|{b_k} - C_b^{\left( 1 \right)}{\left( {x_k^{\left( 1 \right)}} \right)\Big\|^2}\end{equation*}

which is the sum of squared prediction errors of population B activity using population A activity under the RNN model in equation (2). To learn the state and parameters, we minimize this objective using numerical optimization [28]. Once we learn these, we can then use the learned shared states $x_k^{\left( 1 \right)}$ to learn their mapping to the source activity

\begin{equation*}{a_k} = C_a^{\left(1\right)}\left( {x_k^{\left(1\right)}} \right) + {e_k}.\end{equation*}

To do so, we fit the parameter $C_a^{\left( 1 \right)}$ by minimizing the sum of squared prediction error of population A given the learned $x_k^{\left( 1 \right)}$, that is ${L_a} = \mathop \sum \nolimits_k\| {a_k} - C_a^{\left( 1 \right)}{( {x_k^{\left( 1 \right)}})\|^2}$, again using numerical optimization. Together these two optimizations allow us to learn the parameters associated with the shared latent states $x_k^{\left( 1 \right)}$. Appendix A shows how non-shared states $x_k^{\left( 2 \right)}$ can also be learned if desired, though this is not our focus here. Because cross-population dynamics are optimized first under a dedicated objective, the model prioritizes these dynamics and separates them from within-population dynamics. This ensures that the resulting latent states reflect shared dynamics rather than a mixture of the cross-and within- population dynamics.

### 2.3. State estimation

An advantage of CroP-DYNO over prior models of cross-region interactions is its flexibility in extracting latent states either causally or non-causally in time. Furthermore, once training is complete, CroP-DYNO can infer the shared latent states using source activity alone, without the need to access target activity at inference time. Specifically, latent states can be inferred causally using only past data from the source population, facilitating interpretation of directed interactions; they can also be inferred non-causally using all available source population data to improve state estimation. We first describe the causal filtering approach.

#### 2.3.1. Causal CroP-DYNO enables causal state filtering

Once model parameters for the shared dynamics are learned, we can infer the latent states $x_k^{\left( 1 \right)}$ in the test data using causal filtering by recursively running the learned state update equation (equation (1), line 1). The neural activity in populations A and B can then be predicted from the inferred latent states using the learned mapping networks (equation (1), lines 2–3). We note that causal filtering here refers to temporally causal state filtering using only past source activity and should not be interpreted as evidence of directed causal influence between neural populations

#### 2.3.2. Smoothed CroP-DYNO enables non-causal state estimation

Given the noisy nature of neural population activity, it may be desirable to opt for more accurate latent state estimation via non-causal smoothing, rather than the interpretability of causal filtering. To enable this, we extend CroP-DYNO with a bidirectional RNN architecture consisting of a forward and a backward RNN, each with its own set of parameters, that are trained jointly (figure A1).

The forward RNN processes the population A activity ${a_{1:N}}$. The backward RNN is initialized using the final latent state of the forward RNN, and it processes the time-reversed population A activity ${a_{N:1}}$. The forward and backward latent states $x_k^{\mathrm{f}}$ and $x_k^{\mathrm{b}}$ are concatenated at each time step k into a final smoothed state $x_k^{{\mathrm{smooth}}} = \left[ {x_k^{\mathrm{f}};{\text{ }}x_k^{\mathrm{b}}} \right]$ that preserves information from both passes.

Apart from the bidirectional RNN architecture, the training and inference procedures for smoothed CroP-DYNO are identical to those of CroP-DYNO. Because latent state estimation incorporates both past and future observations of population A, the resulting state estimates are non-causal. Neural activity in populations A and B can be predicted by applying the learned mapping networks ${C_a}$ and ${C_b}$, respectively, to the smoothed state. Further theoretical aspects of non-causal smoothing for nonlinear dynamical models of two time series will be discussed in our other ongoing work [59].

### 2.4. Testing the nonlinearity in cross-population model components

CroP-DYNO allows control over which model components are parameterized as nonlinear functions. Specifically, users can manually select which subset of parameters ${A^{{\prime ^{\left( 1 \right)}}}}$, ${K^{\left( 1 \right)}}$, $C_a^{\left( 1 \right)}$, and $C_b^{\left( 1 \right)}{\text{ }}$ are nonlinear during training. Doing so, users can construct candidate models in which different parameters are nonlinear and compare them, thus localizing which components of the cross-population interaction model benefit from nonlinearity and which can be kept linear. This can facilitate interpretability of the cross-population model. In our implementation, nonlinear components are modeled using feedforward neural networks in the form of multilayer perceptrons (MLPs) due to their universal function approximation capabilities [60]. In particular, we used an MLP with a single hidden layer consisting of 128 hidden units.

Alternatively, CroP-DYNO can automatically select an appropriate nonlinearity configuration based on the training data. We refer to this variant as flexible CroP-DYNO. In this approach, for each outer cross-validation fold we perform an inner cross-validation procedure on the training data to select among candidate nonlinearity configurations [28].

Here, we test nonlinearities in the parameters relevant to cross-population prediction of target activity from source activity, which is our major objective (namely ${A^{^{\prime}\left( 1 \right)}}$, ${K^{\left( 1 \right)}}$, and $C_b^{\left( 1 \right)}{\text{ }}$). During training, each of these components may be set to either linear or nonlinear, resulting in a total of eight candidate configurations to train and test. For all nonlinear components, the network architecture is fixed to a single hidden layer with 128 hidden units. For each candidate nonlinearity configuration, we perform two-fold inner cross-validation on the training data and evaluate cross-population prediction performance on the inner held-out validation sets. The configuration yielding the highest cross-population prediction performance on the inner validation sets is then selected. A final model with the selected configuration is then trained on the full training data and evaluated on the held-out test data.

### 2.5. Model assessments

#### 2.5.1. *Evaluating the importance of nonlinearity by comparing to cross-population linear dynamical modeling* (*CroP-LDM)*

To examine the importance of nonlinearity in learning shared cross-population dynamics, we compare CroP-DYNO with CroP-LDM. CroP-LDM uses the same model structure and learning objective as CroP-DYNO (equation (1)) but restricts all model parameters to be linear functions. Under this restriction, the model reduces to a standard linear state-space formulation [22, 61].

Model parameters in CroP-LDM are learned using the same training procedure as in CroP-DYNO, with the objective of accurately predicting population B activity from population A activity. The only difference between CroP-LDM and CroP-DYNO is the form of the model parameters: in CroP-LDM, ${A^{^{\prime}\left( 1 \right)}}$, ${K^{\left( 1 \right)}}$, $C_a^{\left( 1 \right)}$, and are linear functions, whereas in CroP-DYNO they may be nonlinear. As a result, CroP-LDM serves as a linear benchmark that isolates the contribution of nonlinear modeling in capturing cross-population interactions.

#### 2.5.2. Evaluating the importance of prioritized learning by comparing to nonlinear dynamical modeling (NLDM)

To examine the importance of prioritized learning, we compare CroP-DYNO with NLDM without prioritized learning. NLDM uses the same nonlinear RNN model as CroP-DYNO (equation (1), lines 1–2), but it does not prioritize learning of the shared cross-population dynamics through the cross-population prediction objective in equation (3). Instead, NLDM is trained to model the overall dynamics of the source neural population (i.e. population A)—consisting of both the shared cross-population dynamics and other dynamics. Specifically, NLDM describes the activity of population A with a nonlinear RNN (equation (1)) and learns the RNN parameters by minimizing the prediction error of population A activity from its own past activity. This differs from CroP-DYNO, whose objective is to minimize prediction error of population B activity using the past population A activity (equation (3)). Comparing NLDM with CroP-DYNO therefore isolates the effect of prioritized learning of shared latent states through the cross-population prediction objective.

#### 2.5.3. *Comparison with a recent cross-population dynamical model delayed latents across groups* (*DLAG*)

We also compare CroP-DYNO with DLAGs [51], a recent dynamical method that models shared and within-population latent states using Gaussian processes and, similar to CroP-DYNO, allows for inference using only source activity once training is complete. Note that DLAG supports only non-causal latent state inference, whereas CroP-DYNO supports both causal and non-causal inference. Thus, to enable a fair comparison with DLAG, we evaluate CroP-DYNO using its non-causal (smoothed) variant in these comparisons. As DLAG does not aim to prioritize cross-population dynamics, this comparison also highlights the importance of a prioritized learning objective.

### 2.6. Modeling interactions within and across regions

To model interactions within and across brain regions, similar to [22], we sample neural populations in the associated brain regions and then learn the shared cross-population dynamics using our method. Specifically, to model interactions across two regions, we select a neural population from each region and model their shared dynamics with CroP-DYNO. To model interactions within a single region, we instead select two non-overlapping populations from the same region and model their shared dynamics with CroP-DYNO. We note that within-region models capture predictive interactions between the sampled subpopulations and should not be interpreted as characterizing the full recurrent circuitry or direct local connectivity of the region. We make these population selections randomly within each region and repeat multiple times to obtain a robust characterization as follows.

For Monkey I, we randomly sample electrodes from both S1 and M1 to construct four non-overlapping populations: a *source* S1 population, a *target* S1 population, a *source* M1 population, and a *target* M1 population. Each electrode is assigned to at most one population, both within and across repeats, and the population size ranges from 21 to 44 electrodes per population across sessions (mean 32). When the source and target populations are drawn from the same region, the resulting model captures within-region interactions; when they are drawn from different regions, the model captures cross-region interactions.

For Rat S, we randomly split the CA1 and CA3 electrodes into two non-overlapping groups per region, yielding four populations: a *source* CA1 population, a *target* CA1 population, a *source* CA3 population, and a *target* CA3 population. Population sizes ranged from 7 to 12 units depending on the recording session; the population sizes were constant within a session. As above, within-region interactions are modeled when both populations are drawn from the same region, and cross-region interactions are modeled when they are drawn from different regions.

For both subjects, this random population sampling procedure was repeated for each recording session, 2 times for Monkey I and 5 times for Rat S. By repeating across multiple representative populations, this approach reduces sensitivity to electrode selections and yields more robust characterization of the underlying cross-population interactions. For both datasets, we therefore first average performance across repeats within each session-fold to obtain a single representative value per session-fold before computing summary statistics. This yields a total of session-folds for Monkey I (7 sessions × 5 folds) and n=15 session-folds for Rat S (3 sessions × 5 folds).

We evaluate predictive interactions by assessing how well the activity of a given source population predicts the activity of a given target population. For each method and dataset, we fit four models corresponding to all source–target combinations defined above. For Monkey I, these models capture S1 → M1, M1 → S1, S1 → S1, and M1 → M1 interactions. For Rat S, the corresponding models capture CA1 → CA3, CA3 → CA1, CA1 → CA1, and CA3 → CA3 interactions. Together, these configurations allow us to model both cross-region interactions and within-region interactions.

### 2.7. Cross-validated model evaluation

After learning the CroP-DYNO model parameters, we infer latent states in held-out test data and use them to predict the neural activity in the target population. We use five-fold cross-validation to evaluate model performance. Within each fold, we z-score the training and test data by subtracting the mean and normalizing by the standard deviation of the training data. To evaluate the quality of the predicted cross-population dynamics, we compute as our performance metric the cross-validated correlation coefficient (CC) between the true and predicted neural activity in the target population, averaged across neural dimensions.

### 2.8. Selection of latent state dimension

We next describe how we choose the optimal latent state dimension for each model in each session and fold. Using the training data, we first fit models with a latent state dimension ranging from 2 to 32, in powers of 2. The optimal state dimension is then chosen as the smallest value whose training performance is within 2% of the maximum performance achieved across all latent state dimensions. We report test performance using this selected optimal latent state dimension.

### 2.9. Quantifying interaction strengths with partial *R*^2^ metric

To quantify the amount of non-redundant information about a target population $\left( {\mathrm{B}} \right)$ contained in a source population, we use the partial ${R^2}$ metric [22, 62]. Partial ${R^2}$ measures the improvement in predictive performance obtained by including additional inputs in a model. In our setting, the baseline model predicts the activity of the target population using only its own past activity $\left( {{\mathrm{B}} \to {\mathrm{B}}} \right)$. We then compare this to a model that additionally incorporates the past activity of the source population $\left( {{\mathrm{A}},{\mathrm{B}} \to {\mathrm{B}}} \right)$. The partial ${R^2}$ is defined as the relative reduction in the prediction error between these two models, and therefore quantifies the unique contribution of the source population, beyond the target population’s intrinsic predictability.

Formally, to quantify the proportion of variance in population B explained by population A beyond that explained by population B alone, we compute the following partial ${R^2}$ metric:

\begin{equation*}{\mathrm{partial}}\,{R^2} = \frac{{\left({\mathrm{SSE}}\left({\mathrm{B}} \to {\mathrm{B}}\right) - {\mathrm{SSE}}\left({\mathrm{A}},{\mathrm{B}} \to {\mathrm{B}}\right)\right)}}{{{\mathrm{SSE}}\left({\mathrm{B}} \to {\mathrm{B}}\right)}}\end{equation*}

where ${\mathrm{SSE}}\left( {{\mathrm{B}} \to {\mathrm{B}}} \right)$ denotes the sum of squares of the prediction error when only the past of population B is used for prediction, and ${\mathrm{SSE}}\left( {{\mathrm{A}},{\mathrm{B}} \to {\mathrm{B}}} \right)$ denotes the sum of squares of the prediction error when the past of both populations B and A is used for prediction.

### 2.10. Statistical tests

All paired statistical tests in this work were performed using the Wilcoxon signed-rank test. Multiple comparisons were corrected using the Benjamini–Hochberg false discovery rate (FDR) procedure [63].

## 3. Results

### 3.1. Nonlinear modeling and prioritized learning improve the characterization of cross-population dynamics across subjects and brain regions

We first used CroP-DYNO to model cross- and within-region interactions across two species (Monkey I and Rat S) and multiple brain regions (sensory, motor and hippocampus) and evaluate the importance of nonlinearity and prioritized learning in doing so. To characterize cross-region interactions, we modeled the shared dynamics between neural populations from two distinct regions. To characterize within-region interactions, we modeled the shared dynamics between two non-overlapping populations within the same region (section 2.6). In this work, shared dynamics refer to the dynamics in one population that are predictive of the dynamics in another population, potentially reflecting cross-population interactions. We evaluated how well CroP-DYNO identified shared dynamics by assessing its ability to predict the target population’s activity, ${b_k}$, using the shared states, $x_k^{\left( 1 \right)}$, inferred solely from the source population’s activity, ${a_k}$ (section 2.3.1).

To evaluate the importance of nonlinear modeling, we compared CroP-DYNO against CroP-LDM, which is a linear dynamical model with prioritized learning [22]. To assess the importance of prioritized learning of shared dynamics, we compared CroP-DYNO vs NLDM, which learns the same nonlinear RNN model as CroP-DYNO but without the prioritized learning objective in equation (3) (see section 2.2.2).

Across both datasets and all four region-pairs per dataset, CroP-DYNO consistently outperformed NLDM in predicting the target activity across state dimensions (figure 2; green vs purple). This result suggests that the prioritized learning offered by our method is important for learning shared neural dynamics. CroP-DYNO also consistently outperformed CroP-LDM in predicting the target activity across state dimensions (figure 2; green vs pink). This result demonstrates that the incorporation of nonlinearity in the model further improves the learning of shared dynamics. Together, these results are consistent with the presence of nonlinear interactions in cross-population communication and show that CroP-DYNO can capture such structure. Given these results, we next asked whether our method can localize the source of nonlinearity in the cross-population interaction model.

**Figure 2.**
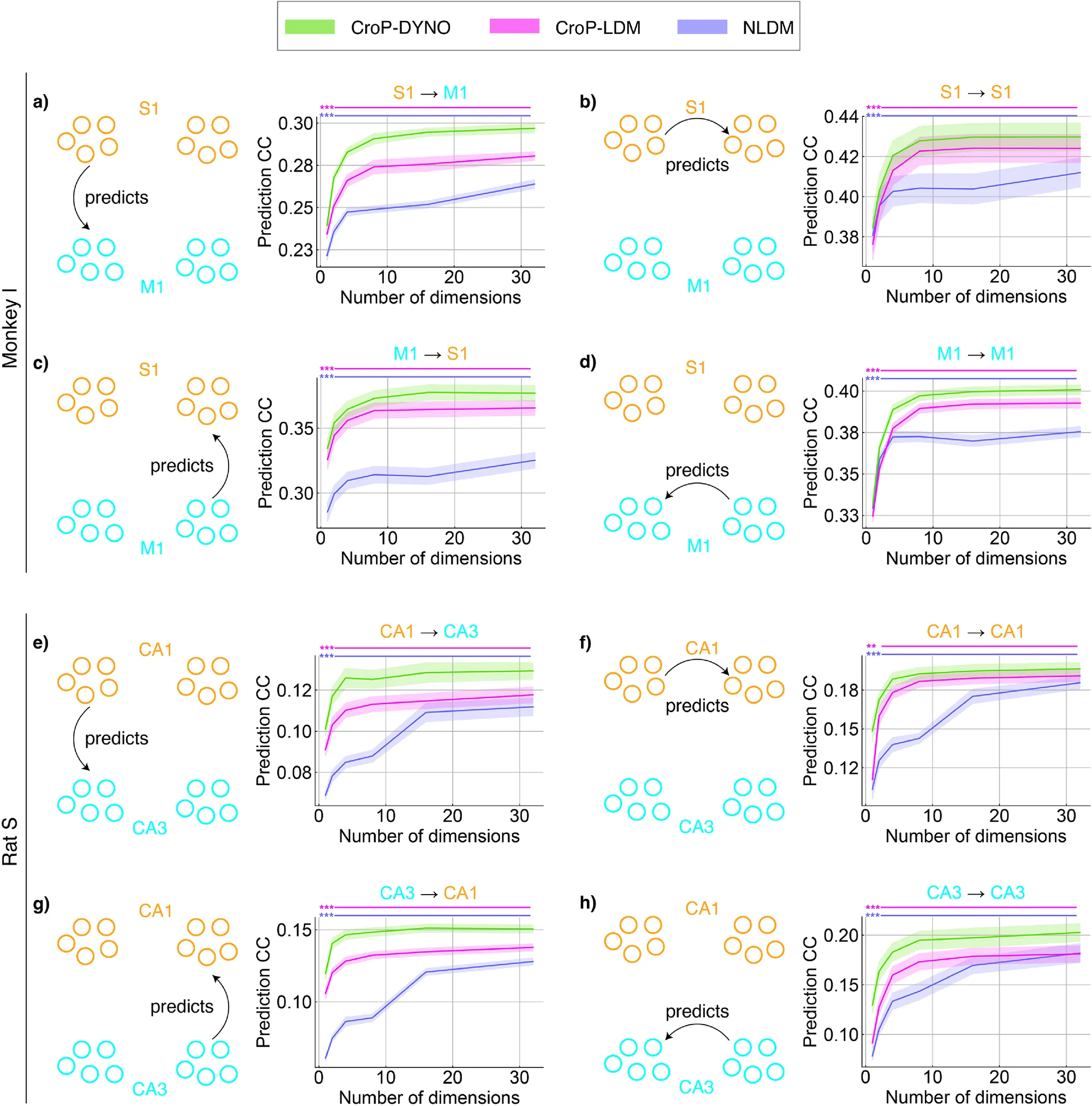
Nonlinearity and prioritized learning in CroP-DYNO are important for accurately predicting the target region’s neural activity from the source region’s neural activity across state dimensions. For each brain region, we split the electrodes into non-overlapping source and target populations of equal size, as illustrated by the icons. (a)–(d) Comparing the cross-validated correlation coefficient (CC) of predicting the target region’s activity from the source region’s activity across latent state dimensions for CroP-DYNO, CroP-LDM (linear prioritized learning), and NLDM (nonlinear non-prioritized learning) for Monkey I. Solid lines show the CC averaged across the datasets, and the shaded areas indicate the standard error of measure, s.e.m. (session-folds) Across latent state dimensions, the statistical significance of a one-sided Wilcoxon signed-rank test, FDR corrected, is shown with a horizontal line and asterisks (purple: CroP-DYNO vs NLDM; pink: CroP-DYNO vs CroP-LDM). Asterisks show the significance level of the comparison (*P < 0.05, **P < 0.005, ***P < 0.0005). (a) S1 → M1 prediction (b) S1 → S1 prediction. (c) M1 → S1 prediction. (d) M1 → M1 prediction. (e)–(h) Same as (a)–(d) for Rat S (n=15 session-folds). (e) CA1 → CA3 prediction. (f) CA1 → CA1 prediction. (g) CA3 → CA1 prediction. (h) CA3 → CA3 prediction.

### 3.2. CroP-DYNO localizes nonlinearity in the model of shared cross-population dynamics

Having shown the presence of nonlinearity in the cross-population data, we next used CroP-DYNO to localize where nonlinear structure arises within the model when fit to these neural recordings. To do this, we compared the cross-population predictive performance of a fully linear CroP-DYNO model (all parameters being linear matrices, section 2.5.1), CroP-DYNO models with single individual nonlinear parameters at different locations (section 2.4), and the flexible nonlinear CroP-DYNO model (section 2.4). CroP-DYNO models with a single nonlinear parameter use a feedforward neural network for that parameter and keep all other parameters linear. Since our focus is on learning the shared cross-population states $x_k^{\left( 1 \right)}$ and their corresponding parameters, we omit the superscript (1) for the remainder of the paper.

#### 3.2.1. Dataset 1: S1 and M1

Prior work suggests that interactions within sensorimotor pathways exhibit nonlinear dynamics [64–66]. In the first dataset, across the four region pairs spanning somatosensory and motor cortices, we found that introducing nonlinearity exclusively in the ${C_b}$ parameter—i.e. in the mapping from the shared cross-population dynamics to target population—was sufficient to achieve high cross-population prediction accuracy (predict target from source). Specifically, models with nonlinearity restricted to ${C_b}$ attained the highest performance among models with individual nonlinear components (figures 3(a)–(d)). Moreover, the CroP-DYNO model with flexible nonlinearity achieved prediction accuracy comparable to that of the ${C_b}$ -only nonlinear model (figures 3(a)–(d), green vs purple). Together, these results suggest that, for interactions within and across somatosensory and motor cortices, the dominant source of nonlinearity can be captured in the ${C_b}$ parameter within the model.

**Figure 3.**
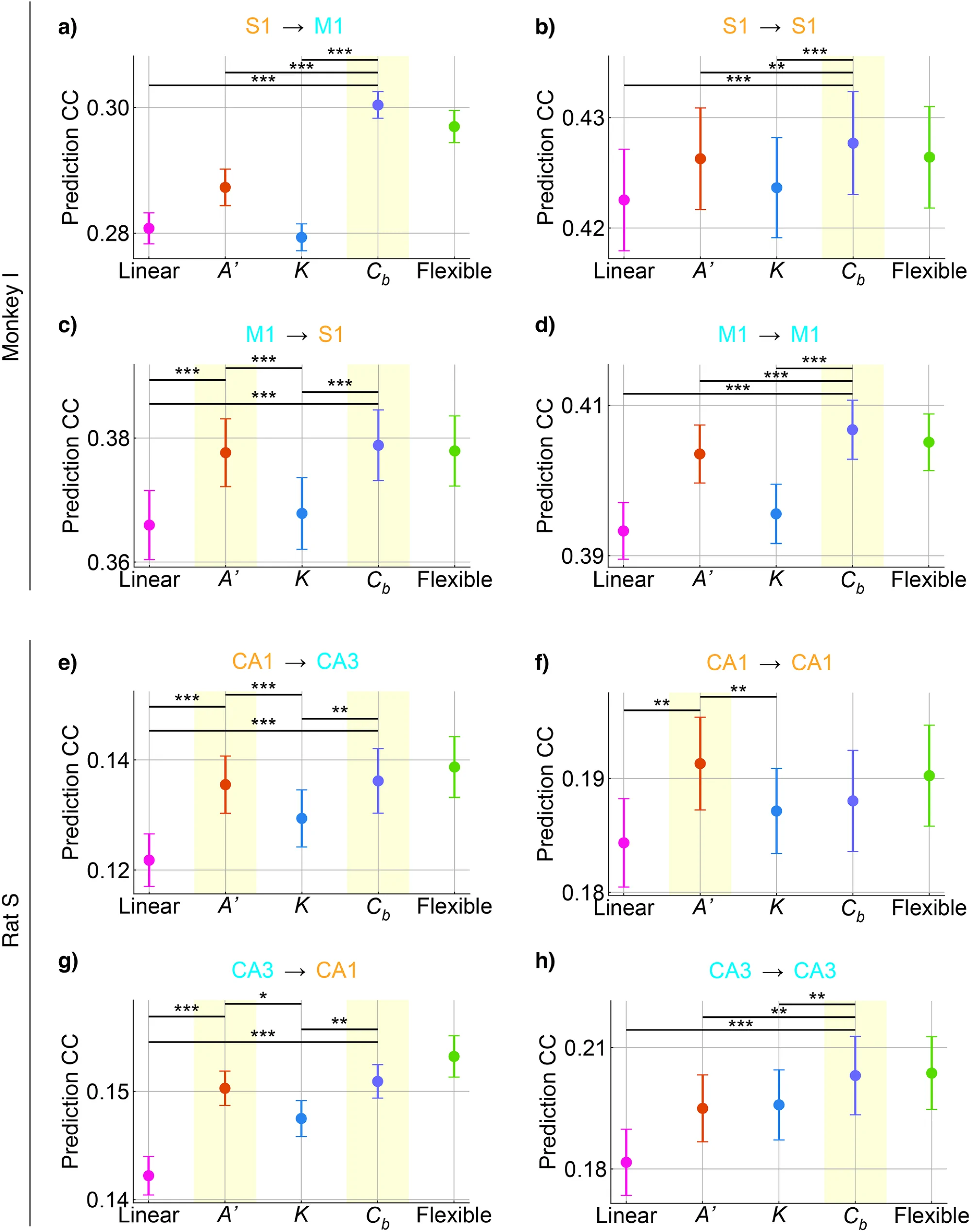
CroP-DYNO localizes nonlinearities within the model of within- and cross-region dynamics. (a)–(d) Cross-validated prediction CC for models with different nonlinearity settings in Monkey I (n=35 session-folds). Models include a fully linear CroP-DYNO model (pink), CroP-DYNO variants with a single nonlinear parameter ($A{^{^{\prime}}}$: orange: blue; ${C_b}$: purple), and a flexibly nonlinear CroP-DYNO model (green). The latent state dimension for each model is chosen as explained in section 2.8. Each point shows the mean prediction accuracy and the whiskers indicate the standard error of measure, s.e.m. Asterisks show significance level for a one-sided Wilcoxon signed-rank test (*P < 0.05, **P < 0.005, ***P < 0.0005). The best-performing method among the linear and single-parameter nonlinear models is highlighted in yellow. The second-best is also highlighted when it is not significantly worse than the best and it significantly outperforms the remaining linear and single-parameter nonlinear methods. (a) S1 → M1 (b) S1 → S1 (c) M1 → S1 (d) M1 → M1 (e)–(h) Same as (a)–(d) for Rat S (n=15 session-folds). (e) CA1 → CA3 (f) CA1 → CA1 (g) CA3 → CA1 (h) CA3 → CA3.

#### 3.2.2. Dataset 2: CA1 and CA3

In the second dataset, across the four region pairs spanning hippocampal subregions, we again used CroP-DYNO to localize the source of nonlinearity in the cross-population communication model. Prior work has established that hippocampal dynamics exhibit nonlinear structure [27, 67]. For cross-region interactions between CA1 and CA3 (i.e. CA1 → CA3 and CA3 → CA1), nonlinearity in either the ${C_b}$ or $A^{\prime}$ parameter yielded similar cross-region prediction accuracy, which was higher than other individual nonlinearities and comparable to CroP-DYNO with flexible nonlinearity figure 3 (e,g). In contrast, for within-region interactions, the location of the nonlinearity was region-specific. Within CA1, nonlinearity in the $A^{\prime}$ parameter yielded the highest accuracy figure 3(f), whereas within CA3, nonlinearity in the ${C_b}$ parameter yielded the highest accuracy figure 3(h).

Together, these results highlight CroP-DYNO’s utility as a tool for localizing nonlinear contributions within a model of cross-region interactions. Furthermore, for cross-region interactions in these specific datasets involving somatosensory, motor and hippocampal regions, our results suggest that having a nonlinear map from the shared cross-population dynamics to the target region (${C_b}$) was largely sufficient for predicting target activity from source activity. Finally, these results indicate that there could also be region-specific structure in the sources of nonlinearity underlying shared neural dynamics.

### 3.3. CroP-DYNO can quantify dominant, biologically consistent interactions between brain regions

We next used CroP-DYNO to quantify the strength of interactions within and across populations of neurons using a normalized measure of cross-population interactions based on partial ${R^2}$ equation (4) [22, 62]. Direct prediction performance (figure 2) is not comparable across different target populations because differences in noise levels and intrinsic predictability can confound interaction strength estimates. Partial ${R^2}$ addresses this issue by quantifying the improvement in predicting a target population’s activity when past activity from a source population is added to a model that already includes the target’s own past activity [22, 62]. This metric quantifies the source population’s contribution beyond what is already predictable from the target itself. We emphasize that the interaction strengths quantified with partial ${R^2}$ do not imply direct causal influence; they instead reflect statistical associations from past source activity to future target activity rather than causality.

#### 3.3.1. Dataset 1: S1 and M1

In our first dataset, we found that the within-region interactions were the strongest, with M1 → M1 exhibiting the highest partial ${R^2}$, followed by S1 → S1. This indicates that a population’s past activity is most predictive of its own future activity, reflecting strong local dynamics (figures 4(a) and (b)). Furthermore, we found that cross-region interactions were weaker than within-region interactions, with M1 → S1 stronger than S1 → M1, though both directions exhibited measurable cross-region prediction as quantified by partial ${R^2}$ (figures 4(a) and (b)). This finding is consistent with known sensorimotor architecture and function; prior studies have shown reciprocal projections between S1 and M1, supporting meaningful prediction in each direction [68–71]. The S1 → M1 interactions convey sensory information [72–75], whereas M1 → S1 interactions transmit predictive or efference copy signals related to motor commands [76–78]. The asymmetric cross-region interactions, with stronger interactions from M1 to S1 (figures 4(a) and (b)), is consistent with prior microstimulation work in NHPs demonstrating stronger effective connectivity from M1 to S1 than in the other direction [66].

**Figure 4.**
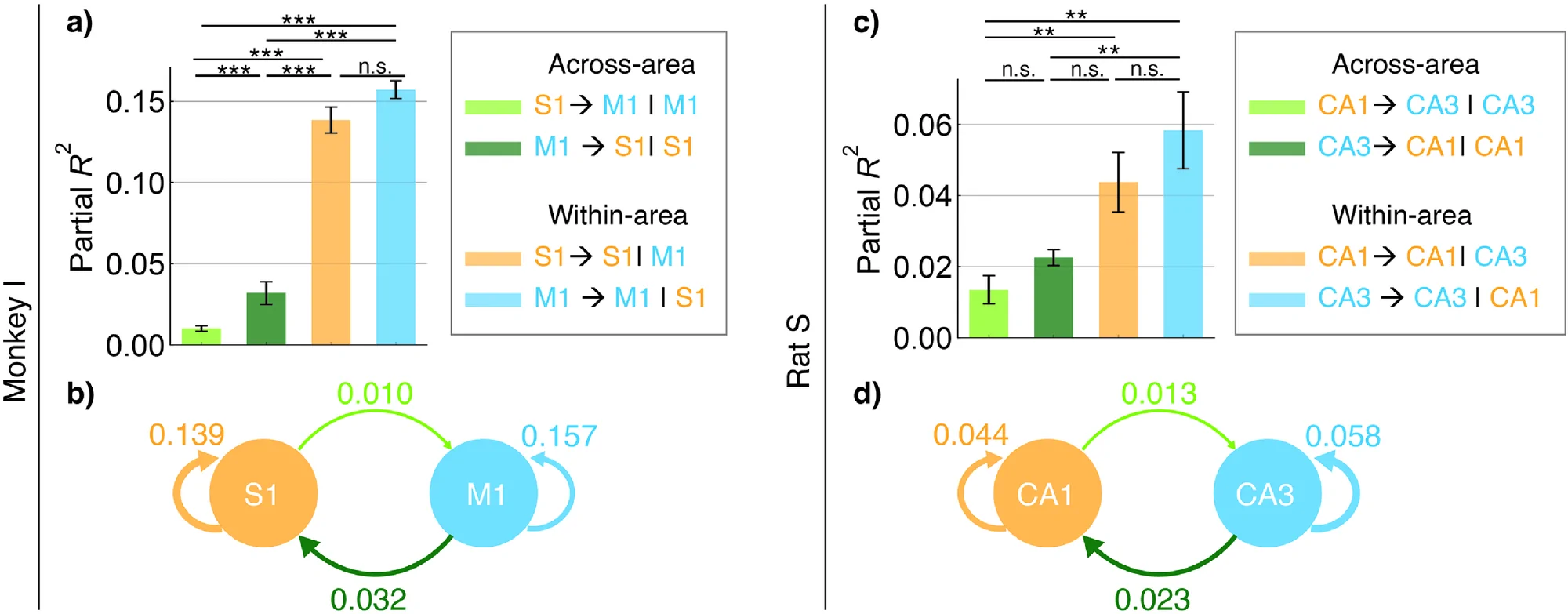
CroP-DYNO quantifies and compares the strength of interactions within and across different brain regions. (a) The mean partial *R*^2^ values obtained by CroP-DYNO for Monkey I at the optimal state dimension for each cross- and within-region scenario. Bars show the mean and whiskers show the standard error of measure, s.e.m. Asterisks are as defined in figure 3. The legend notation ‘S1 → M1 | M1’ indicates the non-redundant information in S1 about M1 beyond that explained by M1 itself, as quantified by the partial *R*^2^ measure. Other legend notations are defined analogously. (b) Graphical representation of the dominant within- and cross-region interactions between S1 and M1 based on the partial *R*^2^ values in a). Line thickness is proportional to the strength of the corresponding interaction. (c) Same as (a) for Rat S across and within CA1 and CA3. (d) Same as (b) for Rat S across and within CA1 and CA3.

Overall, these results indicate that local dynamics dominate the population activity in each cortical area, and that cross-region interactions are present but secondary in strength, with a stronger interaction from motor to sensory cortex.

#### 3.3.2. Dataset 2: CA1 and CA3

In our second dataset, we found that although the cross-region interactions were similar in both directions, consistent with prior work [79], the CA3 → CA1 interaction was slightly stronger than the reverse direction (figures 4(c) and (d)). This finding aligns with the known hippocampal anatomy, in which neurons in CA3 provide a major excitatory projection to CA1 via the Schaffer collateral pathway [80, 81]. This prior evidence that information is predominantly transmitted from CA3 to CA1 through this feedforward circuit aligns with stronger interactions in the CA3 → CA1 direction observed here. The reverse direction (CA1 → CA3), while weaker, may reflect indirect or feedback pathways within the hippocampal circuit (see section 4.4).

We further observed that within-region interactions in CA3 (CA3 → CA3) were stronger than the other three interactions. This finding is consistent with the well-established role of CA3 as an auto-associative network characterized by strong recurrent connectivity, where CA3 pyramidal neurons project back onto other CA3 neurons within the same region [82–84]. Such recurrent circuitry is consistent with strong within-area interactions in CA3. Together, these results align with known hippocampal circuit architecture and demonstrate that CroP-DYNO can recover biologically meaningful interactions both across and within regions.

### 3.4. Non-causal inference with CroP-DYNO

In the analyses above, we used models that predict the activity of a target population using only the past activity of a source population, i.e. perform inference causally in time. This ensures that predictive information in the source precedes activity in the target, which can help with interpretability. Given the noisy nature of neural data, however, incorporating future source activity can enable more accurate estimation—albeit non-causally in time—of the latent states, thereby improving the inference of shared cross-population dynamics and prediction of target activity. We emphasize that by causal and non-causal inference here we refer to inference done either causally over time using past activity alone or non-causally over time using both past and future activity, rather than directed causality.

To enable non-causal inference, we extended the CroP-DYNO learning to support non-causal smoothing via a forward and backward RNN that are trained simultaneously (section 2.3 and figure A1). We refer to this extension as smooth CroP-DYNO. We also used this extension to perform direct comparison with DLAG [51], a recent cross-region dynamical model that supports only non-causal inference of latent states. Similarly to CroP-DYNO, DLAG can extract the shared dynamics and predict the target activity using only source activity at inference time. However, it does not aim to prioritize learning of cross-population dynamics. Across datasets and region pairs, smooth CroP-DYNO outperformed DLAG in predicting target activity (figure 5). This suggests that smooth CroP-DYNO more accurately predicts target population activity due to the better learning of cross-population dynamics.

**Figure 5.**
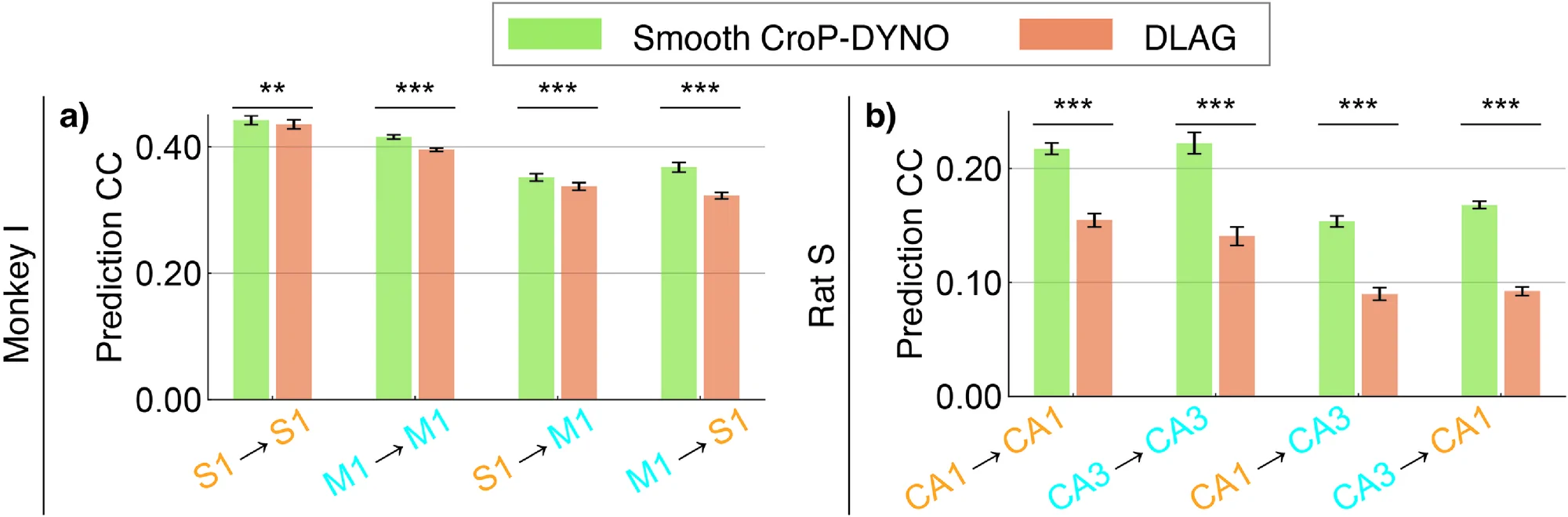
CroP-DYNO with the additional smoothing capability more accurately predicts cross-population activity compared to DLAG. (a) For Monkey I, the mean prediction CC of target activity from source activity for DLAG (orange) and smooth CroP-DYNO (green). Bars show the mean and whiskers show the standard error of measure, s.e.m. Asterisks are as defined in figure 3. (b) Same as (a) for Rat S.

## 4. Discussion

In this work, we introduced CroP-DYNO, a NLDM framework for studying interactions within and across brain regions. By combining a prioritized learning objective with flexible nonlinear state-space modeling using RNNs, CroP-DYNO enables accurate prediction of cross-population activity. Across two different species and four different brain regions, we showed that CroP-DYNO: (1) captures shared cross-population dynamics more accurately than non-prioritized and linear prioritized baselines, showing the importance of nonlinearity and prioritized learning; (2) localizes nonlinear structure in the cross-population model; and (3) yields interaction strengths consistent with known neurophysiology. The consistency of these results across two different datasets, spanning brain regions, task structure, and overt movement versus internal cognitive representations, suggests that CroP-DYNO is applicable across a variety of tasks and neural systems. CroP-DYNO additionally supports inference of cross-population latent states both causally in time and non-causally in time; furthermore, it can infer the cross-population latent states using source activity alone at inference time. When directional interpretability is the goal, causal filtering can be preferable, as it predicts target activity solely based on past source activity. When accurate state estimation is of interest, non-causal smoothing can be better suited, as it performs inference using the full source activity, both past and future.

### 4.1. Both prioritized learning and nonlinear modeling are important for capturing cross-population interactions

We found that both the prioritized learning objective and incorporation of nonlinearity in the CroP-DYNO model are important for accurately modeling cross-population neural interactions.

To demonstrate the importance of prioritized learning, we compared CroP-DYNO against NLDM and DLAG, which lack a cross-population prioritization objective. Comparisons with NLDM demonstrate that even with identical RNN model formulations, NLDM fails to capture cross-population dynamics as accurately since it is not explicitly trained to prioritize them during learning. This highlights an important limitation of NLDM: the latent states learned solely to predict a population’s own dynamics may not necessarily correspond to dynamics that accurately describe cross-population interactions. We further compared to DLAG [51], a recent non-causal cross-region model that does not prioritize cross-population dynamics. CroP-DYNO outperformed DLAG, further supporting the importance of a prioritized learning objective.

To demonstrate the importance of nonlinear modeling, we compared CroP-DYNO against CroP-LDM, a recent linear dynamical model of cross-population interactions with prioritized learning [22]. Across datasets and region-pairs, CroP-DYNO outperformed this linear method; this reveals the insufficiency of linear modeling for fully capturing cross-population dynamics, even when using an objective that prioritizes their learning. These results suggest that nonlinear transformations play a role in shaping cross-population interactions.

### 4.2. CroP-DYNO can localize nonlinearities within the model of cross-population interactions

CroP-DYNO enables systematic localization of nonlinear structure within a model of cross-regional interactions. We note that this localization is at the level of the model and does not directly identify the biological source of nonlinear transformations in the underlying neural circuit. Rather, it indicates where nonlinear structure is most useful for predicting cross-population activity. In our first dataset, consisting of the sensorimotor system, we found that nonlinear structure was predominantly localized to the mapping from the shared cross-population dynamics to the target activity. In our second dataset, consisting of the CA1 and CA3 regions of the hippocampus, nonlinearities could be localized in both this mapping and the latent state dynamics.

These distinctions were identifiable because CroP-DYNO allows individual model parameters to be selectively modeled as linear or nonlinear, enabling targeted and interpretable model comparisons. In contrast, fully linear or fully nonlinear models lack this level of control and may obscure the specific sources of nonlinear structure. At the same time, when nonlinear structure is not supported by the data, CroP-DYNO’s flexible model selection supports identifying linear parametrizations, reducing to a linear model. Together, these results suggest the value of CroP-DYNO’s modular and interpretable design to form testable hypotheses about the nonlinear processing underlying cross-population neural interactions. These insights, in turn, can motivate future targeted causal perturbation experiments to test these hypotheses.

### 4.3. CroP-DYNO supports both causal and non-causal inference and extracts shared dynamics using only source activity at inference time

CroP-DYNO supports inference of latent states either causally or non-causally over time. Causal filtering is important for interpreting directional interaction strengths, as it ensures that predictions of target region activity are based solely on past activity from the source region. However, neural data are inherently noisy, and causal filtering alone may yield less accurate latent state estimates compared with non-causal smoothing that uses both past and future source activity for inference. We thus extended CroP-DYNO to also support non-causal smoothing with a forward–backward smoothing architecture. CroP-DYNO’s causal filtering is more suitable when interpreting directional interaction strengths is of primary interest, while its smoothing is better suited for analyses in which accurate state estimation is of primary interest, such as denoising, visualization of latent dynamics, or other offline analyses. Together, these capabilities make CroP-DYNO a versatile framework that can be adapted to data quality and specific analysis objectives.

Other recent work has developed nonlinear multi-region models with different goals, such as capturing state-dependent communication [85] or disentangling communication from unobserved inputs [86]. CroP-DYNO is complementary to these approaches in that it focuses on prioritized extraction of cross-population dynamics to study directed predictive interactions and localization of nonlinear transformations within specific model components. Another key property of CroP-DYNO is that, although it is trained using simultaneous recordings from both regions, it extracts shared latent states using only source region activity at inference time, enabling prediction of target activity without observing it. This is fundamentally different from some nonlinear multi-region models [85, 86], which process activity from both source and target regions jointly during inference and thus do not support cross-region prediction from source activity alone. Indeed, restricting inference to using source activity alone would likely degrade the performance of these models, as they are designed to jointly leverage activity from both the source and target regions during inference/prediction. This capability to perform state inference using only source activity is essential for studying directed interactions between regions, as it ensures that inferred shared dynamics originate exclusively from the source region. It is also important when target region recordings are unavailable, such as in BCI or deep brain stimulation implants that only cover the source region, or when recordings become degraded due to electrode drift in chronic recordings [43–50]. Our empirical comparisons accordingly focus on methods that, like CroP-DYNO, support this capability, such as DLAG [51] and CroP-LDM [22], and thus can be directly compared in terms of inferring shared dynamics.

### 4.4. Causal filtering and partial R^2^ quantification reveal biologically consistent interaction strengths

Using the causal formulation of CroP-DYNO in conjunction with the partial ${R^2}$ metric, we quantified the relative strength of within- and cross-region interactions while controlling for differences in intrinsic predictability across the target population. Across both datasets analyzed in this study, within-region interactions were consistently stronger than cross-region interactions, reflecting the dominant role of local recurrent dynamics in shaping neural activity for the brain regions and tasks examined.

Importantly, CroP-DYNO recovered cross-region interaction strengths that align with prior neurophysiological evidence. In the sensorimotor dataset, we observed stronger M1 → S1 interactions than S1 → M1 interactions, consistent with predictive or modulatory motor signals influencing sensory cortex alongside weaker, but present, sensory feedback [72–74, 76–78]. In the hippocampal dataset, CA3 → CA1 interactions were stronger than the reverse direction, which may potentially reflect the feedforward Schaffer collateral pathway [80, 81]. Although CA1 → CA3 interactions were weaker, we found that CA1 nevertheless exhibited predictive influence over CA3, as has been previously reported [79]. This predictive relationship may arise from a combination of direct anatomical pathways from CA1 to CA3 as well as shared upstream inputs to both regions. Several anatomical routes could mediate CA1 → CA3 influence, including direct projections via specific interneuron populations [87] or indirect pathways through the entorhinal cortex that connect CA1 to CA3 [88–90]. Additionally, strong CA3 → CA3 interactions were consistent with CA3’s well-established recurrent auto-associative circuitry [82–84]. Together, these biologically consistent findings support the utility of CroP-DYNO as a tool for studying neural interactions.

Despite the findings above, we emphasize that interaction strengths quantified by CroP-DYNO should not be interpreted as evidence of direct causal influence. Although the causal modeling/filtering framework (section 2.3.1) ensures that source activity precedes target activity in time, the resulting predictions reflect correlations rather than functional causality. For example, a shared input driving both populations with different latencies could lead to correlated activity between the populations. More broadly, the latent states learned by CroP-DYNO reflect dynamics in the source population that are predictive of target population activity, but whether these dynamics arise from direct cross-population communication, shared task variables, or common upstream inputs cannot be determined from the modeling alone. Accordingly, any hypotheses regarding the origin or functional causality of the identified interactions should be tested using perturbation-based experiments for example using electrical or optogenetic neurostimulation [8, 38, 49, 91–95].

### 4.5. Future directions

Several directions for future work follow from this study. First, in this work we focused on modeling interactions between two neural populations. Extending CroP-DYNO to simultaneously model interactions among multiple populations could enable characterization of higher-order communication structure and dynamics beyond pairwise interactions. Furthermore, here, we analyzed neural activity in the primate somatosensory (S1) and motor (M1) cortices during a movement task, and in the rodent hippocampal subfields CA1 and CA3 during a working memory task. However, CroP-DYNO makes no assumptions about the specific regions or tasks being studied and can be applied to simultaneous multi-population recordings in other brain regions and during different behavioral paradigms in future work [16–18, 20, 21, 51, 96, 97].

Another future challenge is addressing nonstationarity in modeling cross-population interactions. While prior work has addressed neural nonstationarities using adaptive modeling, these approaches do not explicitly prioritize or isolate cross-population dynamics [98–103]. Extending CroP-DYNO to adaptively track nonstationary interactions represents an important future direction to study changes in cross-population communication over time.

Although our analyses focused on spike count data, future work could validate CroP-DYNO on other neural modalities, including intracranial electroencephalography [104–107], local field potentials [108, 109], or spiking-band power [110, 111]. Additional extensions could incorporate alternative observation models, such as point-process formulations for spiking data [112–117] or multiscale observation models for multimodal recordings [100, 118–126]. Finally, since CroP-DYNO can infer shared dynamics from source activity alone at inference time, it is suited for settings where only a subset of regions is accessible. This includes BCI or deep brain stimulation applications in which implants cover one region but not another, where leveraging cross-population structure could enable principled decoding and modulation of distributed neural activity [49, 50, 99, 110, 127–132]. Incorporating real-time uncertainty quantification into the nonlinear cross-population inference framework is an important direction for future work, particularly for closed-loop neuromodulation applications where robust handling of nonlinear and non-stationary neural dynamics is critical [133, 134].

## Figures and Tables

**Figure A1.**
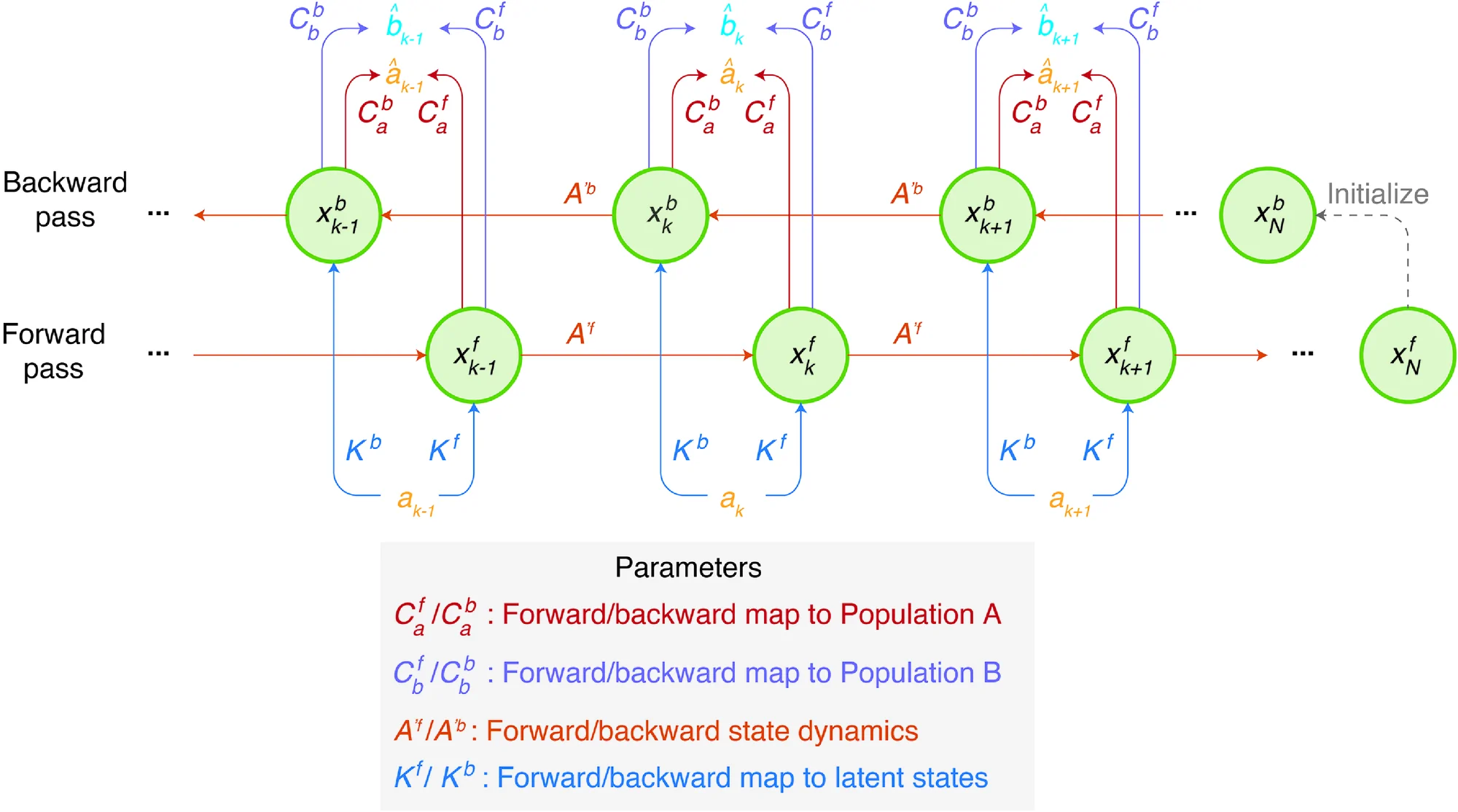
The smoothed CroP-DYNO computation graph for the shared cross-population states, represented as a bidirectional RNN with parameters similar to the mappings introduced in figure 1(a). For simplicity, the superscripts (1) on the states and the parameters indicating their association with the shared cross-population states are omitted. The forward RNN processes the activity of population A (i.e. ${a_{1:N}}$). The backward RNN is initialized with the final latent state of the forward RNN, and it processes the time-reversed activity of population A (i.e. ${a_{N:1}}$). At each time step, the forward and backward latent states, $x_k^{\mathrm{f}}{\text{ }}$ and $x_k^{\mathrm{b}}$, are used to jointly predict the activity of populations A and B via the learned forward and backward mappings.

**Figure A2.**
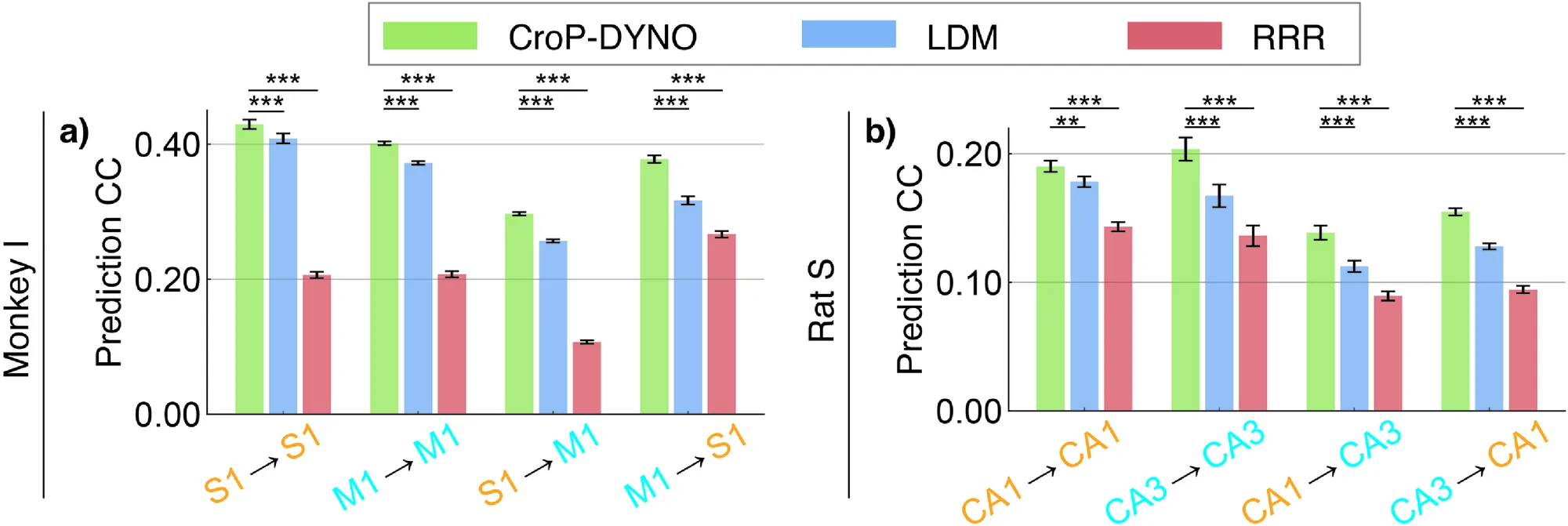
CroP-DYNO more accurately predicts the target region’s neural activity from the source region’s neural activity compared to linear static and non-prioritized linear dynamical baselines. (a) For Monkey I, the mean cross-validated CC of predicting target activity from source activity for CroP-DYNO (green), LDM (linear non-prioritized learning; blue), and RRR (linear static learning; red). Bars show the mean and whiskers show the standard error of measure, s.e.m. Asterisks are as defined in figure 3. (b) Same as (a) for Rat S.

## Data Availability

The main data supporting the results in this study are available within the paper. Raw data from Dataset 1 are publicly available as cited in the paper. Raw data from Dataset 2 are too large to be publicly shared, yet may be made available for research purposes from the corresponding author on reasonable request.

## Code availability statement

The code used to support the results is available at: https://github.com/ShanechiLab/CroP.

## Appendix A Extended model formulation for learning within-population dynamics

Cross-population prioritized dynamical nonlinear interaction model (CroP-DYNO) is designed to primarily extract cross-population dynamics and can be used for cross-region and within-region analyses by learning cross-population dynamics of populations in the same region or in distinct regions, respectively (see section 2.6). Nevertheless, the framework can be extended to additionally characterize within-population dynamics. We describe this optional extension here, which follows a numerical optimization approach similar to [28] for modeling of behaviorally relevant neural dynamics, but instead formulates it for cross-population analyses and extends it beyond its original causal inference to also allow for non-causal inference.

When learning of the within-population dynamics is also of interest, we can expand equation (1) in the form of the below two-section recurrent neural network (RNN):

\begin{align*}\left\{ {\begin{array}{*{20}{l}} {\left[ {\begin{array}{*{20}{c}} {x_{k + 1}^{\left( 1 \right)}} \\ {x_{k + 1}^{\left( 2 \right)}} \end{array}} \right] = \left[ {\begin{array}{*{20}{l}} {{A^{{^{\prime} {\left( 1 \right)}}}}\left( {x_k^{\left( 1 \right)}} \right)} \\ {{A^{{^{\prime} {\left( 2 \right)}}}}\left( {x_k^{\left( 2 \right)}} \right)} \end{array}} \right] + \left[ {\begin{array}{*{20}{c}} {{K^{\left( 1 \right)}}\left( {{a_k}} \right)} \\ {{K^{\left( 2 \right)}}\left( {{a_k},{\text{ }}x_{k + 1}^{\left( 1 \right)}} \right)} \end{array}} \right]} \\ \qquad\quad {{a_k} = C_a^{\left( 1 \right)}\left( {x_k^{\left( 1 \right)}} \right) + C_a^{\left( 2 \right)}\left( {x_k^{\left( 2 \right)}} \right) + {e_k}} \\ \qquad\qquad {{b_k} = C_b^{\left( 1 \right)}\left( {x_k^{\left( 1 \right)}} \right) + { \epsilon _k}} \end{array}} \right..\end{align*}

In this formulation, the latent states $x_k^{\left( 1 \right)}$ represent the shared cross-population dynamics—dynamics of population A that are shared with population B—as written in equation (2), and correspond to the first RNN section. The latent states $x_k^{\left( 2 \right)}$ are in the second RNN section, and they represent dynamics of population A beyond those that are shared with population B (i.e. within-population A dynamics). All the mappings ${A^{^{\prime}\left( 1 \right)}}$, ${A^{^{\prime}\left( 2 \right)}}$, ${K^{\left( 1 \right)}}$, ${K^{\left( 2 \right)}}$, $C_a^{\left( 1 \right)}$, $C_a^{\left( 2 \right)}$, and $C_b^{\left( 1 \right)}{\text{ }}$ are multi-input, multi-output nonlinear functions that parametrize the model.

The learning of the shared cross-population dynamics proceeds using the prioritized cross-population prediction objective in equation (3) as we described in Methods section 2.2.2. Once $x_k^{\left( 1 \right)}$ and its parameters ${A^{{\prime ^{\left( 1 \right)}}}}$, $C_a^{\left( 1 \right)}$, $C_b^{\left( 1 \right)},$ ${K^{\left( 1 \right)}}$ are learned, then we can optionally learn $x_k^{\left( 2 \right)}$ and its parameters ${A^{^{\prime}\left( 2 \right)}},{\text{ }}{K^{\left( 2 \right)}},{\text{ }}$ and $C_a^{\left( 2 \right)}$ of the second RNN section written as:

\begin{align*}\left\{ {\begin{array}{*{20}{c}} {x_{k + 1}^{\left( 2 \right)} = {A^{\prime} }^{\left( 2 \right)}\left( {x_k^{\left( 2 \right)}} \right) + {K^{\left( 2 \right)}}\left( {{a_k},{\text{ }}x_{k + 1}^{\left( 1 \right)}} \right)} \\ {a_k^{\prime} = C_a^{\left( 2 \right)}\left( {x_k^{\left( 2 \right)}} \right) + {e_k}} \end{array}} \right.\end{align*}

Here, $a_k^{\prime}$ refers to the residual population A activity that is not predictable from the already-learned cross-population states $x_k^{\left( 1 \right)}$; that is, $a_k^{\prime} = {a_k} - C_a^{\left( 1 \right)}( {x_k^{\left( 1 \right)}})$. We thus minimize the loss function $L_a^{\left( 2 \right)} = \mathop {\mathop \sum \nolimits }\nolimits_k \|{a_k} - C_a^{\left( 1 \right)}( {x_k^{\left( 1 \right)}}) - C_a^{\left( 2 \right)}{( {x_k^{\left( 2 \right)}} )\|^2}$ to learn $x_k^{\left( 2 \right)}$ and its parameters. This loss uses the already-learned parameter $C_a^{\left( 1 \right)}$ and latent state $x_k^{\left( 1 \right)}$.

In this study, we did not apply this optional second section of the model (i.e. we set ${n_x} = {n_1}$) since our focus was on exclusively extracting cross-population dynamics. Even for within-region analyses, we partitioned each region into non-overlapping subpopulations and modeled their cross-population interactions to study within-region dynamics (Methods section 2.6), similar to prior work [22].

## Appendix B Additional baselines

### B.1 Reduced rank regression (RRR)

As a baseline to assess the benefit of dynamical modeling, we compare CroP-DYNO to RRR, a static method that has been used to characterize interactions across brain regions [20]. RRR relates source and target activity through a linear regression whose coefficient matrix is constrained to be low rank. Because RRR relates source and target activity at a single time step, it does not capture temporal structure in the neural activity.

To match the one-step-ahead prediction used for CroP-DYNO, we predict activity in population B (${n_b}$ electrodes) from the preceding time step of population A (${n_a}$ electrodes). We form $A \in {\mathbb{R}^{\left( {N - 1} \right) \times {n_a}}}$ from source population A’s activity at time steps 1 through N−1, and $B \in {\mathbb{R}^{\left( {N - 1} \right) \times {n_b}}}$ from target population B’s activity at time steps 2 through N. The unconstrained coefficient matrix $W \in {\mathbb{R}^{{n_a} \times {n_b}}}$ is obtained by ordinary least squares:

\begin{align*}{W_{{\mathrm{OLS}}}} = {\left( {{A^T}A} \right)^{ - 1}}{A^T}B.\end{align*}

RRR then imposes a rank constraint, ${\mathrm{rank}}\left( W \right) = m$, where m quantifies the number of source dimensions that are predictive of the target. This constrained solution is obtained from the singular value decomposition of the unconstrained prediction, $A{W_{{\mathrm{OLS}}}} = U{{\Sigma }}{V^T}$, by retaining only its top m principal components ${V_m} \in {\mathbb{R}^{{n_b} \times m}}$:

\begin{align*}{W_{{\mathrm{RRR}}}} = {W_{{\mathrm{OLS}}}}{V_m}V_m^T.\end{align*}

Predicted target activity is then given by ${\hat B_{{\mathrm{RRR}}}} = A{W_{{\mathrm{RRR}}}}$.

### B.2 Linear dynamical modeling (LDM)

As a further baseline, we compare CroP-DYNO with LDM [22], which combines the linearity restriction of CroP-LDM (section 2.5.1) with the non-prioritized training procedure of nonlinear dynamical modeling (NLDM) (section 2.5.2). LDM shares CroP-DYNO’s model structure (equation (1)) but restricts all parameters to linear functions. Like NLDM, its parameters are learned without the cross-population prediction objective in equation (3). Instead of prioritizing the dynamics shared with population B, LDM is fit to explain population A’s own activity. Its parameters are learned by minimizing the prediction error of population A’s activity from its own past, so that the resulting latent states capture the overall dynamics of population A rather than the dynamics specifically shared with population B. Comparing LDM with CroP-DYNO thus reflects the joint contribution of nonlinearity and prioritized learning.
